# gDesigner: computational design of synthetic gRNAs for Cas12a-based transcriptional repression in mammalian cells

**DOI:** 10.1038/s41540-022-00241-w

**Published:** 2022-09-16

**Authors:** Michael A. Crone, James T. MacDonald, Paul S. Freemont, Velia Siciliano

**Affiliations:** 1 Section of Structural and Synthetic Biology, Department of Infectious Disease, Imperial College London, London, United Kingdom; 2 UK Dementia Research Institute Centre for Care Research and Technology, Imperial College London, London, United Kingdom; 3 London Biofoundry, Imperial College Translation and Innovation Hub, White City Campus, 84 Wood Lane, London, United Kingdom; 4 Istituto Italiano di Tecnologia IIT, Department of Synthetic and Systems Biology for Biomedicine, Genoa, Italy

**Keywords:** Molecular biology, Software

## Abstract

Synthetic networks require complex intertwined genetic regulation often relying on transcriptional activation or repression of target genes. CRISPRi-based transcription factors facilitate the programmable modulation of endogenous or synthetic promoter activity and the process can be optimised by using software to select appropriate gRNAs and limit non-specific gene modulation. Here, we develop a computational software pipeline, gDesigner, that enables the automated selection of orthogonal gRNAs with minimized off-target effects and promoter crosstalk. We next engineered a *Lachnospiraceae* bacterium Cas12a (dLbCas12a)-based repression system that downregulates target gene expression by means of steric hindrance of the cognate promoter. Finally, we generated a library of orthogonal synthetic dCas12a-repressed promoters and experimentally demonstrated it in HEK293FT, U2OS and H1299 cells lines. Our system expands the toolkit of mammalian synthetic promoters with a new complementary and orthogonal CRISPRi-based system, ultimately enabling the design of synthetic promoter libraries for multiplex gene perturbation that facilitate the understanding of complex cellular phenotypes.

## Introduction

Mammalian synthetic biology leverages characterized genetic regulatory parts to perform input-output processing algorithms, enabling novel biological functions in eukaryotic cells^1–3^ and tackling fundamental questions underlying biological processes^4–7^. To achieve a comprehensive understanding of cellular phenotypes and functions, new genetic modules (transcription factors, post-transcriptional and translational modulators) are required tools for multi-layered regulation of synthetic networks^8–12^.

Orthogonal transcriptional repressors provide the means to design universal logic circuits, enabling the assembly of tunable and composable devices of increasing complexity^13^. Synthetic transcriptional repressors such as TALE^14^ or zinc-finger proteins^15,16^ fused to effector domains (e.g., KRAB) efficiently down-modulate gene expression, but are limited by slow temporal gene reactivation^17^. Alternatively, transcriptional repressors that directly bind to operator sequences in proximity to the transcription start site and inhibit transcription by steric hindrance have been successfully demonstrated^10,11^.

Catalytically dead Cas9 from *Streptococcus pyogenes* (dSpCas9) as a transcriptional tool to activate^18^ or repress^19^ endogenous and exogenous gene expression, allows transcriptional modulation in a multiplexed and orthogonal fashion, with ground-breaking potential in the biomedical and industrial fields. By means of single guide RNAs (gRNAs) to potentially target nearly any DNA sequence, CRISPR-Cas9 based transcriptional regulation also overcomes the issue of metabolic burden imposed on cells by circuits that simultaneously express a large number of transcription factors to achieve sophisticated regulation^20–22^. However, the common gRNA scaffold sequence prevents the simultaneous use of dCas9 as activator and repressor, limiting possible genetic network designs, and thus hampering their applicability in sophisticated circuits development.

It has been shown that CRISPR-Cas12a nucleases offer several additional features and advantages compared to SpCas9 such as shorter guide RNAs (CRISPR RNAs-crRNAs), RNase processing of multiple crRNAs from a single transcript^23,24^ and the ability to target T-rich protospacer-adjacent motif (PAM) in contrast to the G-rich PAM used by Cas9^25–27^. Transcriptional activators based on catalytically inactive *Lachnospiraceae* bacterium Cas12a (dLbCas12a) fused to synthetic activator VPR, and transcriptional repressors deriving from *Acidaminococcus* sp. BV3L6 (dAsCas12a) fused to Krab repressor domain, have been recently demonstrated^28,29^ in mammalian cells. Moreover, the development of software algorithms to select gRNA with minimized off-target sites by performing exhaustive searches against genomic sequences, has improved the capability to engineer libraries of devices^30–33^, paving the way for genome-wide gene-perturbation screens in mammalian cells, as well as sophisticated design of multilayered networks.

**Fig. 1:**
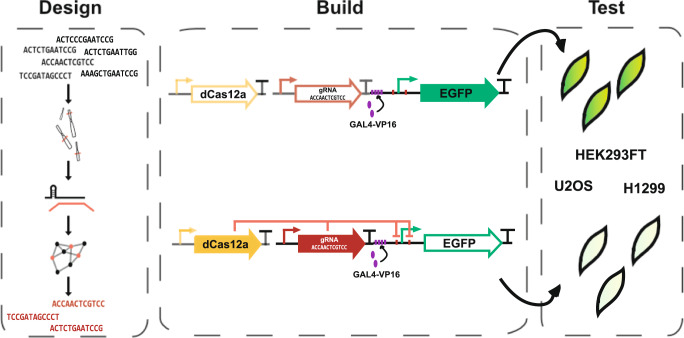
Framework of the study. gDesigner enables the automated selection of orthogonal gRNAs with minimized off-target effects and promoter crosstalk. We generated a library of orthogonal synthetic dCas12a-repressed promoters that downregulates target gene expression by means of steric hindrance of the cognate promoter. We tested the library in HEK293FT, U2OS and H1299 cells lines.

Here, we have developed gDesigner, a software pipeline to select appropriate gRNA sequences and associated synthetic promoters with embedded cognate responsive elements for trans-regulation by dLbCas12a protein (Fig. 1). The software takes an initial pool of generated sequences, and automatically selects the largest orthogonal subset that present minimal similarity, low potential for cross-interactions, and minimal off-targets in the host organism genome.

We have then experimentally demonstrated dLbCas12a-based synthetic transcriptional repressors that inhibit gene expression solely by steric hindrance in different mammalian cell lines (Fig. 1), expanding the CRISPR based tools for genetic cross-regulation and allowing combination of dCas9/dCas12a activators and repressors. We have made this pipeline available at https://github.com/jmacdona/gDesigner.

## Results and discussion

### Generation of synthetic gRNA sequences

An initial pool of synthetic gRNA sequences were generated using R2oDNA Designer, a simulated annealing-based algorithm, previously developed as a general and flexible method to design orthogonal spacer sequences with user-defined constraints^34,35^. Specifically, we designed random sequences according to the following criteria: i) minimal repeats or inverted repeat sequences, ii) minimal BLAST hits to the human genome and iii) no sequences matching eukaryotic transcription factor binding site motifs (according to the HOCOMOCO database^36^), or some common restriction site sequences.

**Fig. 2:**
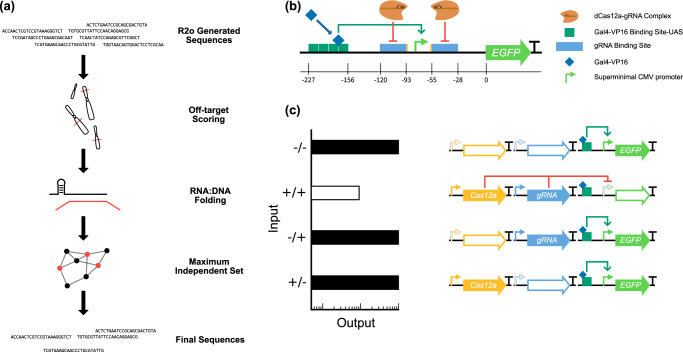
gDesigner pipeline, promoter architecture and system dynamics. **a** Initial sequences are generated using the R2oDNA Designer method. The sequences are subsequently filtered through the gDesigner pipeline (https://github.com/jmacdona/gDesigner) to remove sequences with predicted off-target sites, binding affinities outside a specified range, selecting a final set of orthogonal sequences with minimal predicted cross-interactions. **b** Schematics of the promoter architecture. Gal4-VP16 activates the expression of EGFP, while the binding of Cas12a-gRNA controls its repression. 23 bp gRNA-binding sites with their PAM sequences (5’-TTTN-3’) flank the smCMV promoter. The 5’ binding site is directly adjacent to the TATAA box, and 36 bp downstream the UAS. The 3’ binding site is placed 10 bp downstream of the transcription start site (TSS) and 105 bp downstream of UAS. **c** Schematics of the predicted NAND logic gate. EGFP expression is repressed in the presence of hdLbCas12a/gRNA-promoter complex.

This initial pool of gRNA sequences was then fed into our new synthetic gRNA-screening pipeline, gDesigner. The pipeline automatically eliminates sequences with potential off-target hits in specified genomes^37^, and spacer sequences with estimated hybrid gRNA-spacer:cognate-DNA binding site affinities outside a defined range of values^38,39^. In the last step, gDesigner retrieves an orthogonal set of gRNA sequences with no pairwise cross-interactions between different gRNA species below a specified minimum free energy, using a simple graph-theoretic algorithm to find the maximum independent set (Fig. 2a, Supplementary Table 2).

### Optimization of binding site orientation to maximize repression

**Fig. 3:**
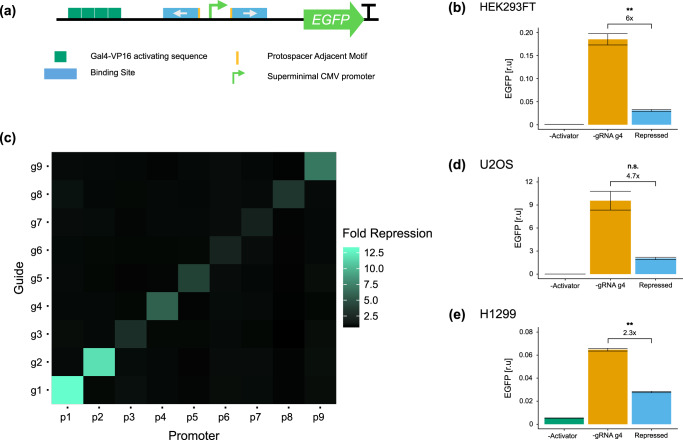
Test of hdLbCas12a functionality and orthogonality in mammalian cell lines. **a** Design of the best-repressed promoter configuration. Binding sites facing opposite directions flank the smCMV promoter. The 5’-TTTN-3’ PAM is proximal to either side of smCMV. **b** EGFP levels in HEK293FT cells in absence of activator as indication of promoter leakiness (−Activator) or gRNA (−gRNA g4), or upon binding of hdLbCas12a/gRNA complex (Repressed). Data represent geometric mean and standard deviation of means of EGFP MEFL normalized by mKate expression for cells expressing >2 × 10^4^ MEFL of the transfection marker mKate for at least 3 pooled replicates. **c** Orthogonality matrix of synthetic gRNA/promoters. Repression levels were measured between all combinations of hdLbCas12a/gRNAs and promoters in order to determine levels of repression (with cognate pairs) and crosstalk (with non-cognate pairs). Fold repression is calculated by dividing the normalized geometric mean of EGFP MEFL of unrepressed cells (−gRNA) by the normalized geometric mean of EGFP MEFL of repressed cells (+gRNA), where EGFP MEFL geometric mean is normalized by mKate expression for cells expressing >2 × 10^4^ MEFL of transfection marker mKate for at least 2 replicates. Of note, −gRNA samples included hdLbCas12a along with Gal4-VP16 and reporter encoding plasmids to take into account the burden imposed to the cells. **d**, **e** Test of hdLbCas12a/g4 transcription factor/promoter pair-induced repression was tested in U2OS and H1299 cell lines. Promoter output was measured in absence of activator (−Activator), gRNA (-gRNA g4) or in presence of hdLbCas12a/g4 (Repressed). Data represent geometric mean and standard deviation of means of EGFP MEFL normalized by mKate expression for cells expressing >2 × 10^4^ (H1299) or >5 × 10^3^ MEFL (U2OS) MEFL of transfection marker mKate for at least 2 replicates. Statistical significance, calculated using the Student’s *t* test, is denoted by asterisks (where ** means *p* < 0.01).

To test synthetic gRNA sequences generated by gDesigner, we engineered a promoter architecture framework that allows easy drop-in of synthetic gRNA binding sites and used single point mutations to catalytically deactivate a mammalian codon-optimized Cas12a from *Lachnospiraceae bacterium ND2006* (hdLbCas12a). Repressible promoters are composed of Gal4-VP16-responsive 4x UAS operator sequences, and gRNA binding sites upstream and flanking a super minimal CMV (smCMV) promoter, to regulate the expression of enhanced green fluorescent protein, EGFP (Fig. 2b). Binding of the Cas12a-gRNA complex to the cognate promoter prevents Gal4-VP16-induced transcriptional activation (Fig. 2c) computing a Boolean NAND logic gate. Similar to previous studies^19^ we first investigated the correlation between gRNA binding sites flanking the TATA box directionality and repression efficiency. We designed and tested four promoter configurations with gRNAs facing inwards, outwards, 5’, or 3’ direction in HEK293FT cells (Supplementary Fig. 1a). We observed stronger hdLbCas12a repression either when gRNA binding sites face outwards the TATA box, or in 5’ direction (Supplementary Fig. 1b, Fig. 3a, b). For the next set of experiments, we placed gRNA binding sites to face outwards relative to the TATA box.

### Testing orthogonality of repression of generated synthetic gRNAs and minimal promoters

To maximize the activation-repression ratio we tested increasing levels of Gal4-VP16 along with fixed concentration of the other components of the repressor and used the optimized condition (GAL4-VP16:Cas12a:gRNA ratio: 1:1, 5:10) for subsequent tests (Supplementary Fig. 2). Next, we tested hdLbCas12a-dependent repression in HEK293FT cells, using a library of nine computationally designed synthetic gRNA/promoters which were co-transfected along the GAL4-VP16 and in the presence or absence of gRNA (Fig. 3c, Supplementary Table 1). Also, by co-transfecting all combination of reporters and transcription factors into HEK293FT cells (9 × 9 matrix), we show that our repressors are largely orthogonal (Fig. 3c, Supplementary Fig. 3). Promoters/gRNAs that exhibited different repression activity in HEK293FT were also tested in U2OS (g1-g4) (Fig. 3d, Supplementary Fig. 5) and H1299 (g4) cell lines (Fig. 3e). Interestingly, U2OS cells show similar repression activity to that observed in HEK293FT cells (repression induced by g2 and g1 > g4 > g3) albeit with overall different fold of activation (Supplementary Fig. 5) and repression (Fig. 3b–e, Supplementary Figs. 3 and 4), likely due to cell-dependent differences in the pool of available transcription factors and co-factors^40^.

Our library provides a further expansion to the toolbox of transcriptional regulators that can be layered in complex networks. Minimal cross talk in synthetic promoters is a critical feature of genetic circuits. We demonstrate that gDesigner allows the design of a library of nine gRNA/promoters pairs that show extensive orthogonality. In contrast to recently developed dCas12a based repressors that downregulated endogenous gene expression via fusion to Krab domain, our synthetic repressors effectively repress solely by steric hindrance, potentially overcoming issues related to slow temporal reactivation^17^.

A disadvantage to the design of complex regulatory networks is the size limit of the DNA for transient expression or integration, as well as the burden that multiple transcription factors in layered circuits impose to the host cells. CRISPR based transcriptional regulation can overcome this limitation since any circuit topology can be encoded by a single transcriptional repressor along with multiple gRNAs to simultaneously downregulate multiple genes. Here, we provide a proof-of-principle study that dLbCas12a can down-modulate gene expression in a similar way to that of dCas9 in human cells^19^. This should enable the engineering of multilayered networks of increasing complexity. Taken together, our method should facilitate a broad range of applications for biomedical interventions in human cells.

## Method

### Synthetic gRNA design and binding site selection

The new software pipeline implemented as a Python module, was developed to rapidly screen synthetic orthogonal gRNA sequences that conform to a given set of required constraints. Source code and installation instructions are available at https://github.com/jmacdona/gDesigner. The user inputs an initial pool of FASTA-formatted candidate synthetic gRNA sequences (here, we used the R2oDNA Designer simulated annealing algorithm but sequences from any source are possible), a user-defined constraints/settings file, and a paths file to instruct the pipeline where software dependencies are installed.

This pipeline was composed of four steps: (i) initial sequence pool generation using the R2oDNA Designer algorithm, (ii) off-target screening using OFF-FINDER, (iii) RNA:DNA hybrid free energy screening using MELTING (iv) calculation of all-against-all pairwise minimum free energies (MFE) using PairFold^41^ and determination of the largest orthogonal set of gRNA sequences by retrieving the maximum independent set. The initial pool of DNA sequences (step (i)) was generated using the previously published simulated annealing-based algorithm, R2oDNA Designer, which eliminates repeats above a defined length, inverted repeats above a defined length and other disallowed sequences^34^. Sequences for LbCas12a were generated in the form of a repeat-spacer (TAATTTCTACTAAGTGTAGATN_23_). Certain restriction sites, promoter sequence motifs, and eukaryotic transcription factor binding site motifs from the HOCOMOCO database^36^ were disallowed. Sequences that generated BLAST hits against the *E coli* BL21, DH10β and W3110, *B. subtilis*, *S. cerevisiae* and *H. sapiens* genomes as well a catalog of IGEM parts were excluded below a certain e-value cutoff. The variable region (Nx) of the generated sequences were designed to have a 60 °C DNA melting temperature.

Cas-OFFinder^37^ was used to search both Human and *E. coli* genomes for potential off-target sequences of up to 7 mismatches with the PAM sequence 5’-TTTN-3’. A previously described off-target scoring scheme for Cas9^30^ was adapted to score for Cas12a by using the mismatch matrix from a recent publication^23^. The scoring scheme calculates the score of each individual off-target sequence (Eq. (1)) and then sums the scores together to give a final quantitative score (Eq. (2)). Sequences with scores below 0.8 were eliminated.

Equation 1 (single mismatch score):

1 $$\mathop {\prod}\limits_{{{{\mathrm{e}}}} \in {{{\mathcal{M}}}}} {\left( {1 - W\left[ e \right]} \right)} \times \frac{1}{{\left( {\frac{{22 - {{{\bar{\mathrm d}}}}}}{{22}} \times 4 + 1} \right)}} \times \frac{1}{{{{{\mathrm{n}}}}_{{{{\mathrm{mm}}}}}^2}}$$

where e is the mismatch position, ${{{\mathcal{M}}}}$ is the mismatch matrix with scores according to mismatch position, ${{{\bar{\mathrm d}}}}$ is the mean pairwise distance and n_mm_ is the number of mismatches.

Equation 2 (aggregate score):

2 $${{{\mathrm{S}}}}_{{{{\mathrm{guide}}}}} = \frac{{100}}{{100 + \mathop {\sum}\nolimits_{{{{\mathrm{i}}}} = 1}^{{{{\mathrm{n}}}}_{{{{\mathrm{mm}}}}}} {{{{\mathrm{S}}}}_{{{{\mathrm{hit}}}}}\left( {{{{\mathrm{h}}}}_{{{\mathrm{i}}}}} \right)} }}$$

Where S_guide_ is the aggregate score for the guide RNA and S_hit_(h_i_) is the score for each individual genomic hit.

Equation 3 (mismatch matrix):

3 $${{{\mathcal{M}}}} = \left[\begin{array}{ccccccccccccccccccccccc} {0.63} & {0.59} & {0.78} & {0.70} & {0.67} & {0.72} & {0.61} & {0.43} & {0.31} & {0.23} & {0.13} & {0.32} & {0.21} & 0 & {0.35} & {0.21} & {0.08} & {0.25} & {0.45} & {0.38} & {0.34} & {0.36} & 0 \end{array} \right]$$

In an attempt to maintain similar repression strengths between different CRISPRi promoters, only gRNA spacer sequences with predicted hybrid gRNA-spacer/cognate-DNA binding free energies with a specified range were retained. Hybrid DNA-RNA free energies were calculated using MELTING^38,39^.

Finally, all-against-all pairwise minimum free energies (MFE) were calculated for all synthetic gRNA sequences using PairFold^41^. The largest orthogonal set of synthetic gRNA sequences was determined by retrieving the maximum independent set using the Python graph library NetworkX^42^. An undirected graph was constructed, where each node corresponded to a gRNA sequence. The nodes were connected by edges if the sequences had Smith-Waterman scores above a user defined value, sub-sequence matches above a user defined length or MFEs below a user defined value. The maximum clique of the complement graph then gives the maximum independent set. This was carried out using the *find_cliques* function of NetworkX which implements existing algorithms^43–45^.

### Plasmid assembly

Circular Polymerase Extension Cloning (CPEC) was used for the construction of most of the plasmids used in this paper. Assembly conditions and primer design followed recommendations from Quan et al.^46^.

pMC085-pcDNA3.1-hdLbCas12a was created using site directed mutagenesis and CPEC. pcDNA3.1-hLbCas12a (Addgene #69988) was amplified in three fragments using primers to obtain the catalitycally inactive mutant LbCas12a (D832A). Fragments were digested with DpnI (NEB), gel purified using the Zymoclean™ Gel DNA recovery kit (Zymo Research) and reassembled in a thermocycler (Annealing: 55 °C, Extension: 5:00, Cycles: 15). Positive clones were then confirmed by DNA sequencing.

Drop-out constructs pMC102-Prom-RFP and pMC088-U6-Cas12agRNA-RFP were created using CPEC to allow for Type-IIs restriction site cloning of promoter binding sites and gRNAs respectively.

pMC102-Prom-RFP backbone (containing UAS binding site, mammalian EGFP, bacterial origin of replication, Kanamicyn resistance and polyA signal) was PCR amplified from pLS1-BoxCDGC_2xKMet_^47^ and the dropout was amplified from an Integrated DNA Technologies gBlock containing a constitutively expressed RFP.

pMC088-U6-Cas12agRNA-RFP backbone (containing U6 promoter, bacterial origin of replication and ampicillin resistance) was PCR amplified from pSIREN_U6-shRNA_FF5-CMV-iRFP (courtesy of Dr. Wroblewska), and dropout was PCR amplified from the same Integrated DNA Technologies gBlock containing constitutively expressed RFP. Fragments were then digested with DpnI (NEB), gel purified using the Zymoclean™ Gel DNA recovery kit (Zymo Research) and assembled in a thermocycler using the following conditions: Annealing: 55 °C, Extension: 2:30 (pMC102-Prom-RFP) 2:00 (pMC088-U6-Cas12agRNA-RFP), Cycles: 15.

Colonies were screened according to their phenotype (red color) upon transformation and confirmed with DNA sequencing.

gRNA constructs were created using TypeIIs assembly^48^. Annealed, phosphorylated oligonucleotides with the LbCas12a repeat sequence and specified spacer sequence, were combined in a reaction with T4 DNA Ligase (NEB), BspMI (NEB), pMC088-U6-Cas12agRNA-RFP, Ligase Buffer (NEB) and Bovine Serum Albumin (NEB). Reactions were then thermocycled for 30 cycles of 37 °C for 5:00 followed by 16 °C for 10:00 as described previously^49^. Colonies were screened according to their phenotype (white color) and confirmed with DNA sequencing.

Promoter constructs were created using TypeIIs assembly^48^. Annealed phosphorylated oligonucleotides with flanking binding sites and a central superminimal CMV promoter (TATATAAGCAGAGCTCGTTTAGTGAACCGTCAGATCGC) were ligated to respective plasmidic backbones in a reaction mix including with T4 DNA Ligase (NEB), BpiI (Thermo Fisher Scientific), pMC102-Prom-RFP, Ligase Buffer (NEB) and Bovine Serum Albumin (NEB). Reactions were then thermocycled for 30 cycles of 37 °C for 5:00 followed by 16 °C for 10:00 as described previously^49^. Colonies were screened according to their phenotype (white color) and confirmed with DNA sequencing.

Transformations were performed in E. coli DH5α cells cultured at 37 degrees in Lysogeny Broth, supplemented with the appropriate antibiotic (kanamycin 50 μg/ml, carbenicillin 100 μg/ml) (Sigma).

All plasmids were sequence verified (Eurofins) using appropriate sequencing primers (Supplementary Table 3). Sequence verified transformants were grown in lysogeny broth and stored as glycerol stocks at −80 °C in 20% glycerol. For transfections, plasmids were prepared using the QIAfilter plasmid midi kit (Qiagen) following the manufacturer protocol.

### Cell culture

U2OS and HEK293FT cell lines were cultured in Dulbecco’s Modified Eagle Medium (DMEM) (Thermo Fisher Scientific) and H1299 cells were maintained in Roswell Park Memorial Institute Medium (RPMI-1640) (Thermo Fisher Scientific). Media was supplemented with 10% Fetal Bovine Serum (Thermo Fisher Scientific), Penicillin-Streptomycin (Thermo Fisher Scientific), L-Glutamine (Thermo Fisher Scientific) and MEM Non-Essential Amino Acids (Thermo Fisher Scientific) and cells were grown at 37 °C in a 5% CO_2_ incubator.

### Transfections

All cells were transfected using a “fast forward” protocol according to manufacturer instructions. Cells were trypsinized and plated immediately before adding plasmid/liposome complexes. All cell lines were transfected in 24 well plates with an initial cell number of 2 × 10^5^ cells per well.

A total of 540 ng of DNA was transfected: 20 ng gRNA-responsive promoter, 60 ng hdLbCas12a, 400 ng U6-gRNA, 40 ng Gal4VP16, pL-A2 to 540 ng.

HEK293FT cells were transfected using Lipofectamine 3000 (Thermo Fisher Scientific). H1299 cells were transfected using Lipofectamine 2000 (Thermo Fisher Scientific), and U2OS cells were transfected using FuGENE6 (Promega Corporation) following manufacturer’s recommendations. 1 ml of media was added to cells 24 h post-transfection.

For flow cytometer analysis, 48 h post transfection media was removed and cells were washed with PBS before being trypsinized and resuspended in DMEM without phenol red. Cells were then transferred to flow cytometry tubes on ice for sample processing. Cells were analyzed using a BD LSR Fortessa™ flow cytometer.

### Inclusion and ethics statement

Research was conducted in compliance with the Global Code of Conduct.

## Quantification and statistical analysis

### Flow cytometry and data analysis

Cells were analyzed with LSR Fortessa flow cytometer, equipped with 488 and 561 nm lasers (BD Biosciences). We collected 10,000–20,000 events per sample and fluorescence data were acquired with the following cytometer settings: 488 nm laser and a 525/20 nm (H1299 and U2OS) or 530/30 nm (HEK293FT) bandpass filter for EGFP, 561 nm laser and a 610/20 nm (H1299 and U2OS) or 660/20 nm (HEK293FT) bandpass filter for mKate. The population of live cells was selected according to FSC/SSC parameters (Supplementary Fig. 6 and Supplementary Table 4). Data analysis was performed with Flowjo and TASBE using Rainbow Calibration Particles (8 peaks, Spherotech)^50^.

Flow cytometry data was converted from arbitrary units to compensated MEFL (Molecules of Equivalent Fluorescein) using the TASBE characterization method^41^. The TASBE method uses a strong constitutively expressed fluorophore, which serves as both a transfection marker and an indicator of relative circuit copy count. An affine compensation matrix is computed from single positive and blank controls. FITC measurements are calibrated to MEFL using RCP-30-5-A beads (SpheroTech) and mappings from other channels to equivalent FITC are computed from co-transfection of constitutively expressed mKate, controlled by a CMV promoter on its plasmid.

Cells were manually gated during TASBE analysis to select viable cells according to their Side Scatter (SSC-A) and Forward Scatter (FSC-A) characteristics. Single cells were then selected using their Forward Scatter (FSC-H and FSC-W) properties.

Fold repression was calculated by dividing the geometric mean of EGFP MEFL (normalized by the MEFL of the mKate transfection marker) of unrepressed control cells (transfected with the EGFP reporter, Gal4-VP16 and hdLbCas12a expression plasmids) by the transfection marker ormalized geometric mean of EGFP MEFL of unrepressed cells (transfected with plasmids encoding the EGFP reporter, Gal4-VP16, hdLbCas12a and gRNA). Based on the observed constitutive fluorescence distributions, a transfection marker threshold was selected for each data set, below which data were excluded as being too close to the non-transfected population while also retaining a significant number of cells: 5 × 10^3^ MEFL for U2OS, 1.26 × 10^4^ MEFL for H1299 and 2 × 10^4^ MEFL for HEK293FT data sets. Fold repression for a range of transfection marker thresholds are show in Supplementary Figs. S3 and S4(b). High outliers are removed by excluding all bins without at least 100 data points. Population geometric statistics are computed over this filtered set of data. All experiments included at least 2 technical replicates and error bars indicate standard deviation. Variance for all groups is generally similar: any differences are reflected in the displayed standard deviation.

## Supplementary information

SI

## Data Availability

Requests for datasets should directed to the Lead Contact, Velia Siciliano (velia.siciliano@iit.it).

## Code availability

Code is open source and publicly available from Github (https://github.com/jmacdona/gDesigner).
