# Supplementary material for: gDesigner: computational design of synthetic gRNAs for Cas12a-based transcriptional repression in mammalian cells
Source: NPJ Syst Biol Appl. 2022 Sep 16;8:34. doi: 10.1038/s41540-022-00241-w (PMC9481559; doi:10.1038/s41540-022-00241-w)
Supplement: Supplementary file 1 — SI [file 41540_2022_241_MOESM1_ESM.pdf]

Supplementary information

gDesigner: computational design of synthetic gRNAs for Cas12a-based transcriptional repression in mammalian cells

Michael Crone[2,3,4], James T. MacDonald[2]*, Paul S. Freemont[2,3,4]* and Velia Siciliano[1,2]*

Author affiliations:

[1]Istituto Italiano di Tecnologia, Department of Synthetic and Systems Biology for Biomedicine, Genoa, Italy
[2]Imperial College London, Department of Infectious Disease, Section of Structural and Synthetic Biology, London, United Kingdom
[3]UK Dementia Research Institute Centre for Care Research and Technology, Imperial College London, London, UK
[4]London Biofoundry, Imperial College Translation and Innovation Hub, White City Campus, 84 Wood Lane, London, UK

*Correspondence should be addressed to VS (velia.siciliano@iit.it), JTM (j.macdonald@imperial.ac.uk), or PSF (p.freemont@imperial.ac.uk)

Supplementary Table 1: gRNA sequences generated by gDesigner

Supplementary Table 2: Properties of the gRNAs selected by gDesigner and used in this manuscript.

Supplementary Table 3: List of primers and oligos to create and sequence constructs used in this manuscript.

Supplementary Table 4: Vertices for viable cell and single cell gating for HEK293FT, U2OS and H1299 cell lines.

Supplementary Figure 1: Test of promoter binding site orientation.

Supplementary Figure 2: Activation/Repression optimization

Supplementary Figure 3: Library of gRNA/promoter repression in HEK293FT cells.

Supplementary Figure 4: Test of hdLbCas12a functionality and orthogonality in U2OS cells.

Supplementary Figure 5: Gal4VP16 transcriptional activation.

Supplementary Figure 6: Flow Cytometry Gating Strategy

gRNA ID	Sequence Number	Sequence
1	541	ACCAACTCGTCCGTAAAGGGTCT
2	1226	TCGTGAAGCAACCCTGCGTATTG
3	1271	TCCGATAGCCCTGAAGCGACAAT
4	1674	TCGTCGGATAAGCCGTAACTCGT
5	120	TCTGTCCGTGAACCACCGTAAGA
6	512	TCCTCGTGCTCGCTGGTCAATAA
7	1367	TCAACTATCCAGAGCGTTCGGCT
8	1608	TGTGCGTTATTCCAACAGGAGCG
9	1675	ACCCGACAATCGTCCTCTGAAGT

Supplementary Table 1. gRNAs sequences generated by gDesigner. gRNA ID identifies gRNAs tested in HEK293FT, U2OS and H1299 cell lines. Sequence Number refers to the initial identification number of putative gRNA sequences during R2oDNA sequence generation that subsequently undergo a further selection according to user-specific constraints.

ID	Gibbs F (kJ)	Kd F (e-21)	Gibbs R (kJ)	Kd F (e-21)	Offtarget F	Number of Genomic Targets F	Offtarget R	Number of Genomic Targets R
1	*-127064.8*	*0.398560391425*	*-125769.3*	*0.658680221594*	*0.922088264041*	*509*	*0.921181017589*	*487*
2	*-121535.7*	*3.40151680561*	*-123354.4*	*1.68026534275*	*0.91097916801*	*620*	*0.937065065548*	*670*
3	*-129454.1*	*0.157798289521*	*-126386.3*	*0.518517136359*	*0.942018626341*	*600*	*0.905664190681*	*974*
4	*-122773.7*	*2.10463091941*	*-123071.1*	*1.87538133161*	*0.987867362223*	*155*	*0.985303834952*	*124*
5	*-121646.1*	*3.25896504751*	*-126537.0*	*0.489083607261*	*0.874305054017*	*755*	*0.88544016907*	*785*
6	*-121886.0*	*2.96945779105*	*-129155.4*	*0.177177099406*	*0.902210116165*	*829*	*0.845837763566*	*1287*
7	*-123868.8*	*1.37640312142*	*-127955.7*	*0.282133074467*	*0.916618810264*	*632*	*0.933885192459*	*528*

8	*-122612.2*	*2.24065414283*	*-122277.9*	*2.5507956324*	*0.898260716591*	*802*	*0.905996139393*	*812*
9	*-121552.3*	*3.3796906223*	*-127993.3*	*0.278049187768*	*0.917809277441*	*720*	*0.871750582156*	*638*

Supplementary Table 2. Properties of the gRNAs selected by gDesigner and used in this manuscript. The calculated Gibbs Free Energy, Kd, Offtarget score and the number of Genomic Targets are displayed for each gRNA in both the forward (F) and the reverse (R) orientation. All gRNAs have Gibbs Free Energy -130 000 < x < -120 000 and Offtarget scores > 0.8. The number of Genomic Targets is for the allowed number of mismatches as a part of the offtarget scoring (7).

**dLbCas12a**	
LbCas12a fwd	CTATGTGATCGGCATCGCTAGGGGCGAGCGC
LbCas12a rev	CTCGCCCCTAGCGATGCCGATCACATAGGGGTTATCG

**gRNA Cloning Vector**	
hU6 pSIREN Fwd	GCCTTTCTGCGTTTATACACCTGCGACTTTTTTTtcgatgcaaatgattctatcctaaaagacca
hU6 pSIREN Rev	GACTGAGCTAGCCGTCAACACCTGCGTACccggtgtttcgtcctttccacaagatatataaagccaag
hU6 Cas12a RFP Fwd	GTACGCAGGTGTTGACGGCTAGCTCAGTC
hU6 Cas12a RFP Rev	GTCGCAGGTGTATAAACGCAGAAAGGCCC

**Promoter Cloning Vector**	
BbsI CMV_Fwd	TAGAGTGTCTTCGTTGACGGCTAGCTCAGTCCTAGG
BbsI CMV_Rev	TCCAAGGTCTTCGTATAAACGCAGAAAGGCCCACCC
CMV BB_Fwd	TGGGCCTTTCTGCGTTTATACGAAGACCTTGGATCCAATTCGACaccggtcgc
CMV BB_Rev	GGACTGAGCTAGCCGTCAACGAAGACACTCTAGAGTCTCCGCTCGGAGGACAG

**Sequencing Primers**	
hU6 seq primer	GACTATCATATGCTTACCGTAACTTGAAAGTATTTC
Seq Promoter	atgcattagttattaatGCTCCGAATTTCTCGACAGATC

**Promoter Primers**		
smCMV	superminimal CMV fwd	TATATAAGCAGAGCTCGTTTAGTGAACCGTCAGA
	superminimal CMV rev	GCGATCTGACGGTTCACTAAACGAGCTCTGCTTA

Orientation 1		
1226	smCMV Lb1226_11 fwd	TAGATTTATCGTGAAGCAACCCTGCGTATTG
	smCMV Lb1226_11 rev	TATACAATACGCAGGGTTGCTTCACGATAAA
	smCMV Lb1226_12 fwd	TCGCCAATACGCAGGGTTGCTTCACGATAAA
	smCMV Lb1226_12 rev	TCCATTTATCGTGAAGCAACCCTGCGTATTG
1674	smCMV Lb1674_11 fwd	TAGATTTATCGTCGGATAAGCCGTAACTCGT
	smCMV Lb1674_11 rev	TATAACGAGTTACGGCTTATCCGACGATAAA
	smCMV Lb1674_12 fwd	TCGCACGAGTTACGGCTTATCCGACGATAAA
	smCMV Lb1674_12 rev	TCCATTTATCGTCGGATAAGCCGTAACTCGT
Orientation 2		
541	smCMV Lb541_21 fwd	TAGAAGACCCTTTACGGACGAGTTGGTtaaa
	smCMV Lb541_21 rev	TATAtttaACCAACTCGTCCGTAAAGGGTCT
	smCMV Lb541_22 fwd	TCGCtttaACCAACTCGTCCGTAAAGGGTCT
	smCMV Lb541_22 rev	TCCAAGACCCTTTACGGACGAGTTGGTtaaa
1226	smCMV Lb1226_21 fwd	TAGACAATACGCAGGGTTGCTTCACGAtaaa
	smCMV Lb1226_21 rev	TATAtttaTCGTGAAGCAACCCTGCGTATTG
	smCMV Lb1226_22 fwd	TCGCtttaTCGTGAAGCAACCCTGCGTATTG
	smCMV Lb1226_22 rev	TCCACAATACGCAGGGTTGCTTCACGAtaaa
1271	smCMV Lb1271_21 fwd	TAGAATTGTCGCTTCAGGGCTATCGGAtaaa
	smCMV Lb1271_21 rev	TATAtttaTCCGATAGCCCTGAAGCGACAAT
	smCMV Lb1271_22 fwd	TCGCtttaTCCGATAGCCCTGAAGCGACAAT
	smCMV Lb1271_22 rev	TCCAATTGTCGCTTCAGGGCTATCGGAtaaa
1674	smCMV Lb1674_21 fwd	TAGAACGAGTTACGGCTTATCCGACGAtaaa
	smCMV Lb1674_21 rev	TATAtttaTCGTCGGATAAGCCGTAACTCGT
	smCMV Lb1674_22 fwd	TCGCtttaTCGTCGGATAAGCCGTAACTCGT
	smCMV Lb1674_22 rev	TCCAACGAGTTACGGCTTATCCGACGAtaaa

120	smCMV Lb120_21 fwd	TAGATCTTACGGTGGTTCACGGACAGAtaaa
	smCMV Lb120_21 rev	TATAtttaTCTGTCCGTGAACCACCGTAAGA
	smCMV Lb120_22 fwd	TCGCtttaTCTGTCCGTGAACCACCGTAAGA
	smCMV Lb120_22 rev	TCCATCTTACGGTGGTTCACGGACAGAtaaa
512	smCMV Lb512_21 fwd	TAGATTATTGACCAGCGAGCACGAGGAtaaa
	smCMV Lb512_21 rev	TATAtttaTCCTCGTGCTCGCTGGTCAATAA
	smCMV Lb512_22 fwd	TCGCtttaTCCTCGTGCTCGCTGGTCAATAA
	smCMV Lb512_22 rev	TCCATTATTGACCAGCGAGCACGAGGAtaaa
1367	smCMV Lb1367_21 fwd	TAGAAGCCGAACGCTCTGGATAGTTGAtaaa
	smCMV Lb1367_21 rev	TATAtttaTCAACTATCCAGAGCGTTCGGCT
	smCMV Lb1367_22 fwd	TCGCtttaTCAACTATCCAGAGCGTTCGGCT
	smCMV Lb1367_22 rev	TCCAAGCCGAACGCTCTGGATAGTTGAtaaa
1608	smCMV Lb1608_21 fwd	TAGACGCTCCTGTTGGAATAACGCACAtaaa
	smCMV Lb1608_21 rev	TATAtttaTGTGCGTTATTCCAACAGGAGCG
	smCMV Lb1608_22 fwd	TCGCtttaTGTGCGTTATTCCAACAGGAGCG
	smCMV Lb1608_22 rev	TCCACGCTCCTGTTGGAATAACGCACAtaaa
1675	smCMV Lb1675_21 fwd	TAGAACTTCAGAGGACGATTGTCGGGTtaaa
	smCMV Lb1675_21 rev	TATAtttaACCCGACAATCGTCCTCTGAAGT
	smCMV Lb1675_22 fwd	TCGCtttaACCCGACAATCGTCCTCTGAAGT
	smCMV Lb1675_22 rev	TCCAACTTCAGAGGACGATTGTCGGGTtaaa
Orientation 3		
1226	smCMV Lb1226_11 fwd	TAGATTTATCGTGAAGCAACCCTGCGTATTG
	smCMV Lb1226_11 rev	TATACAATACGCAGGGTTGCTTCACGATAAA
	smCMV Lb1226_22 fwd	TCGCtttaTCGTGAAGCAACCCTGCGTATTG
	smCMV Lb1226_22 rev	TCCACAATACGCAGGGTTGCTTCACGAtaaa
1674	smCMV Lb1674_11 fwd	TAGATTTATCGTCGGATAAGCCGTAACTCGT

	smCMV Lb1674_11 rev	TATAACGAGTTACGGCTTATCCGACGATAAA
	smCMV Lb1674_22 fwd	TCGCtttaTCGTCGGATAAGCCGTAACTCGT
	smCMV Lb1674_22 rev	TCCAACGAGTTACGGCTTATCCGACGAtaaa
Orientation 4		
1226	smCMV Lb1226_21 fwd	TAGACAATACGCAGGGTTGCTTCACGAtaaa
	smCMV Lb1226_21 rev	TATAtttaTCGTGAAGCAACCCTGCGTATTG
	smCMV Lb1226_12 fwd	TCGCCAATACGCAGGGTTGCTTCACGATAAA
	smCMV Lb1226_12 rev	TCCATTTATCGTGAAGCAACCCTGCGTATTG
1674	smCMV Lb1674_21 fwd	TAGAACGAGTTACGGCTTATCCGACGAtaaa
	smCMV Lb1674_21 rev	TATAtttaTCGTCGGATAAGCCGTAACTCGT
	smCMV Lb1674_12 fwd	TCGCACGAGTTACGGCTTATCCGACGATAAA
	smCMV Lb1674_12 rev	TCCATTTATCGTCGGATAAGCCGTAACTCGT

**gRNA Primers**		
**Code**		
541	Lb541 gRNA hU6 fwd	CCGGTAATTTCTACTAAGTGTAGATACCAACTCGTCCGTAAAGGGTCT
	Lb541 gRNA hU6 rev	AAAAAGACCCTTTACGGACGAGTTGGTATCTACACTTAGTAGAAATTA
1226	Lb 1226 guide hu6 Fwd	CCGGTAATTTCTACTAAGTGTAGATTCGTGAAGCAACCCTGCGTATTG
	Lb 1226 guide hu6 Rev	AAAACAATACGCAGGGTTGCTTCACGAATCTACACTTAGTAGAAATTA
1271	Lb1271 gRNA hU6 fwd	CCGGTAATTTCTACTAAGTGTAGATTCCGATAGCCCTGAAGCGACAAT
	Lb1271 gRNA hU6 rev	AAAAATTGTCGCTTCAGGGCTATCGGAATCTACACTTAGTAGAAATTA
1674	Lb 1674 guide hu6 Fwd	CCGGTAATTTCTACTAAGTGTAGATTCGTCGGATAAGCCGTAACTCGT
	Lb 1674 guide hu6 Rev	AAAAACGAGTTACGGCTTATCCGACGAATCTACACTTAGTAGAAATTA
120	Lb 120 guide hu6 Fwd	CCGGTAATTTCTACTAAGTGTAGATTCTGTCCGTGAACCACCGTAAGA

	Lb 120 guide hu6 Rev	AAAATCTTACGGTGGTTCACGGACAGAATCTACACTTAGTAGAAATTA
512	Lb 512 guide hu6 Fwd	CCGGTAATTTCTACTAAGTGTAGATTCCTCGTGCTCGCTGGTCAATAA
	Lb 512 guide hu6 Rev	AAAATTATTGACCAGCGAGCACGAGGAATCTACACTTAGTAGAAATTA
1367	Lb 1367 guide hu6 Fwd	CCGGTAATTTCTACTAAGTGTAGATTCAACTATCCAGAGCGTTCGGCT
	Lb 1367 guide hu6 Rev	AAAAAGCCGAACGCTCTGGATAGTTGAATCTACACTTAGTAGAAATTA
1608	Lb 1608 guide hu6 Fwd	CCGGTAATTTCTACTAAGTGTAGATTGTGCGTTATTCCAACAGGAGCG
	Lb 1608 guide hu6 Rev	AAAACGCTCCTGTTGGAATAACGCACAATCTACACTTAGTAGAAATTA
1675	Lb 1675 guide hu6 Fwd	CCGGTAATTTCTACTAAGTGTAGATACCCGACAATCGTCCTCTGAAGT
	Lb 1675 guide hu6 Rev	AAAAACTTCAGAGGACGATTGTCGGGTATCTACACTTAGTAGAAATTA

Supplementary Table 3. List of primers and oligos to create and sequence constructs used in this manuscript.

**Cell Line**	**Viable Cells Gating Vertices**	**Single Cell Gating Vertices**
HEK293FT	(50000,20000),(200000,20000),<br>(200000,200000),(50000,200000)	(120000,40000),(120000,110000),<br>(80000,110000),(80000,40000)
U2OS	(10000,1000),(80000,1000),<br>(80000,40000),(10000,40000)	(80000,6000),(120000,6000),<br>(120000,22000),(80000,22000)
H1299	(10000,1000),(50000,1000),<br>(50000,50000),(1000,50000)	(80000,6000),(125000,6000),<br>(125000,23000),(80000,23000)

Supplementary Table 4. Vertices for viable cell and single cell gating for HEK293FT, U2OS and H1299 cell lines.

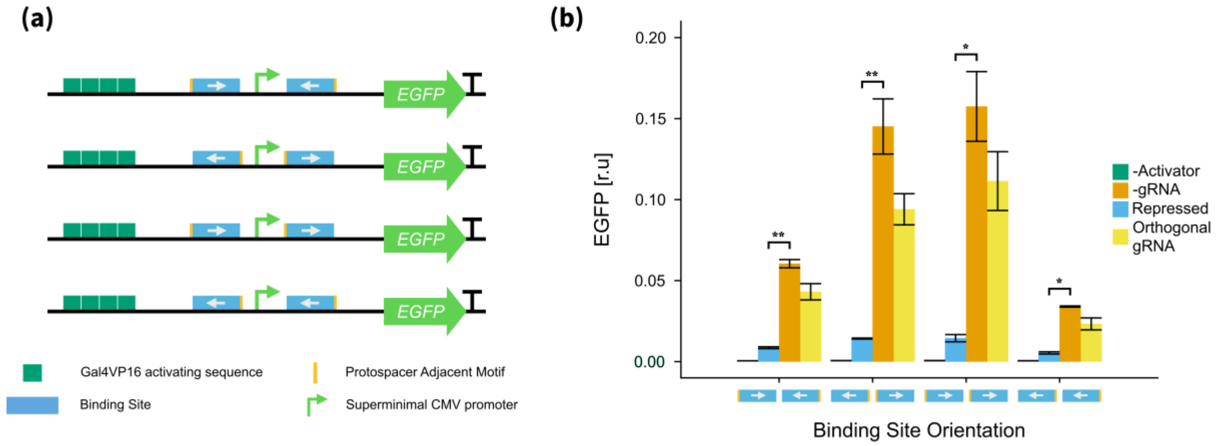

Supplementary Figure 1. Test of promoter binding site orientation. **(a)** Design of different promoters with binding sites orientated inward, outward, 5' and 3' direction. **(b)** Binding site orientation characterisation in HEK293FT cells. The promoter output was measured in each orientation for four different conditions: no Gal4VP16 (-Activator), no gRNA (-gRNA), with cognate gRNA (Repressed) and with gRNA (Orthogonal gRNA). Data represent geometric mean and standard deviation of means of EGFP MEFL normalised by mKate expression for cells expressing > $2x10^4$ MEFL of transfection marker mKate for at least 2 replicates. Statistical significance, calculated using the Student's t-test, is denoted by asterisks (where ** means $p<0.01$).

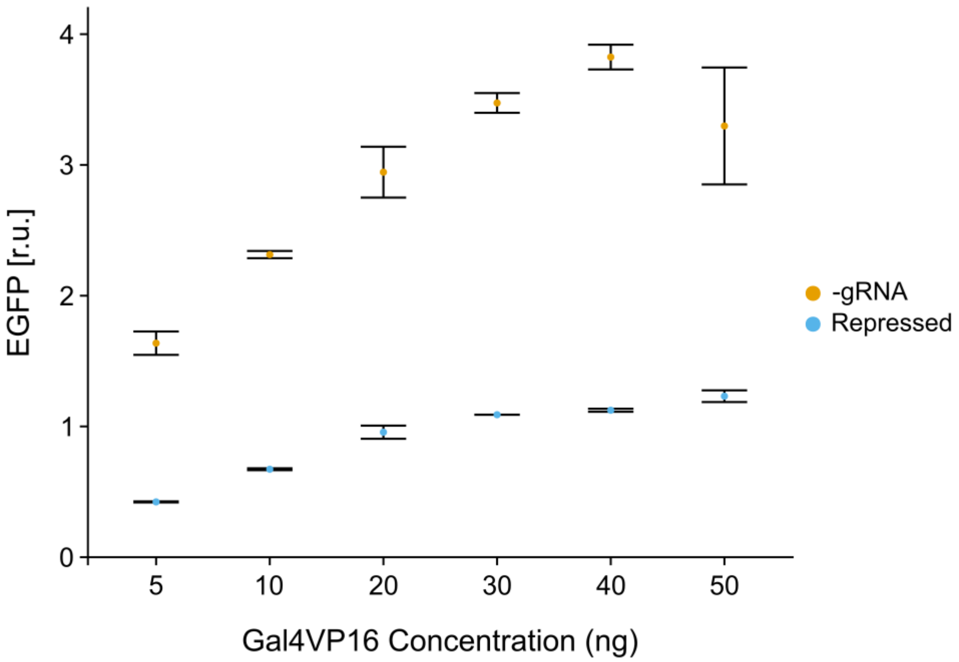

Supplementary Figure 2. Activation/Repression optimization. Increasing concentration of Gal4-VP16 (range 5 to 50 ng) encoding plasmid were co-transfected in HEK293FT cells along with fixed amounts of the other components of the synthetic repression system (+gRNA) or in absence of gRNA (-gRNA). Data represent geometric mean and standard deviation of means of EGFP normalised by mKate for cells expressing >1x10^3 of transfection marker mKate for *n* = 2 replicates.

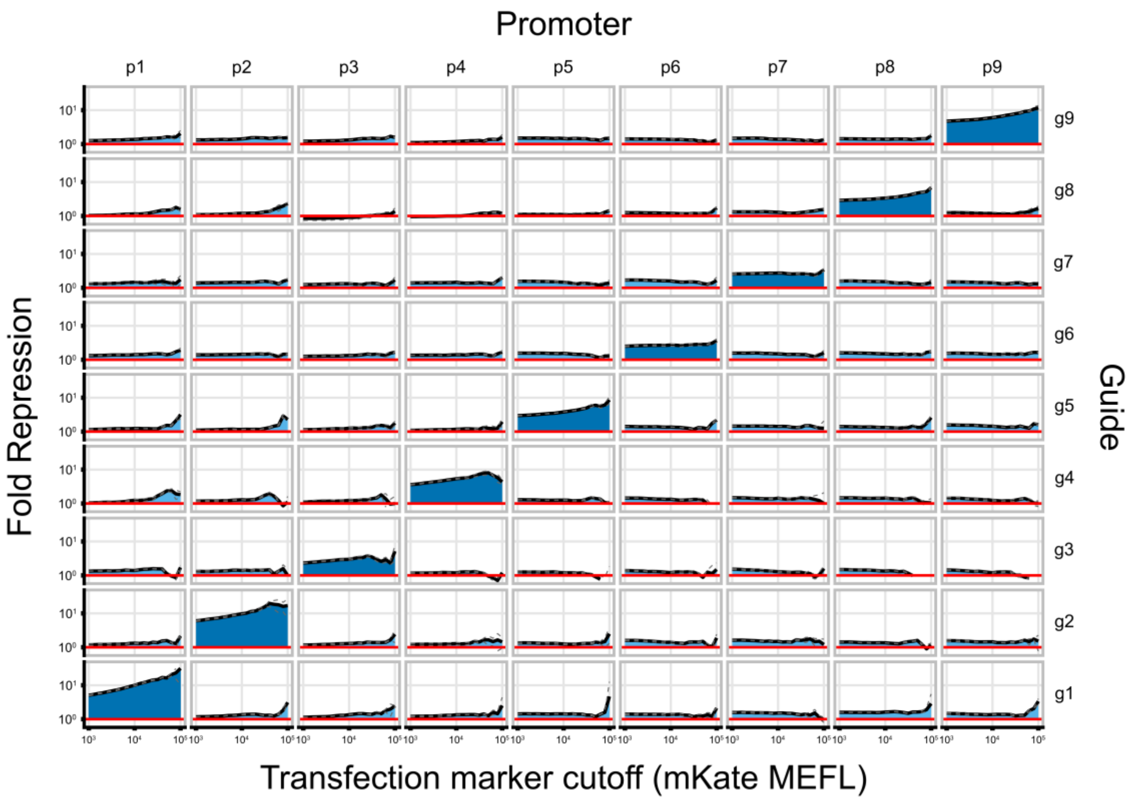

Supplementary Figure 3. Library of gRNA/promoter repression in HEK293FT cells. gRNA-Promoter library was co-transfected in HEK293FT cells in presence or absence of associated gRNAs, or of orthogonal gRNAs. Fold repression (black line) is calculated by dividing the geometric mean of EGFP MEFL (normalised by the MEFL of the mKate transfection marker) of unrepressed control cells (transfected with EGFP reporter, Gal4-VP16 and hdLbCas12a expression plasmids) by the transfection marker normalised geometric mean of EGFP MEFL of repressed cells (transfected with plasmids encoding EGFP reporter, Gal4-VP16, hdLbCas12a and gRNA) for at least 2 replicates at different minimum MEFL cut-offs of the mKate transfection marker. hdLbCas12a was transfected along with Gal4-VP16 and reporter encoding plasmids in the unrepressed control to account for any additional burden imposed to the cells by exogenous plasmid expression. Reference fold repression of 1 is represented by the red line. The dashed grey lines represent standard error.

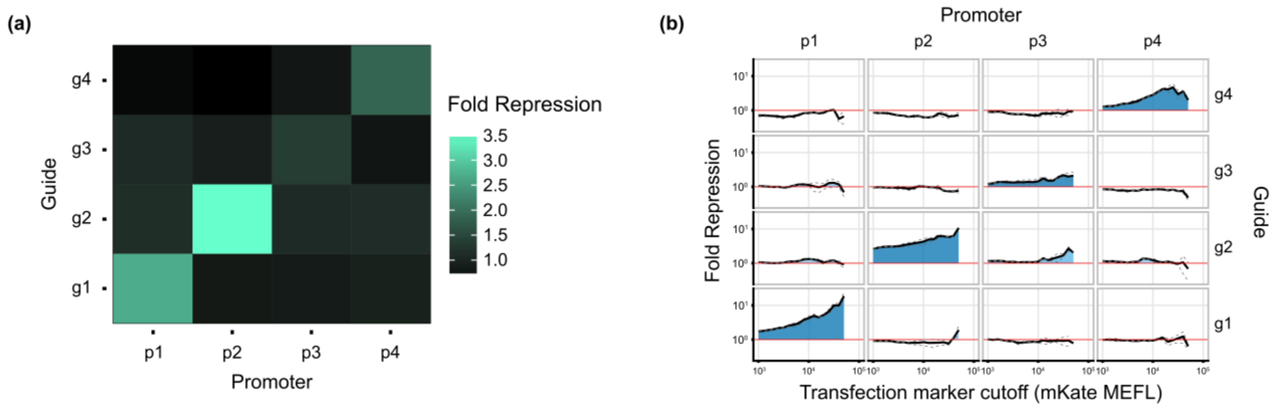

Supplementary Figure 4. Test of hdLbCas12a functionality and orthogonality in U2OS cells. (a) Orthogonality matrix of gRNAs g1-g4 in U2OS cells. Fold repression is calculated by dividing the transfection marker normalised geometric mean of the EGFP MEFL of unrepressed control cells by the normalised geometric mean of gRNA repressed cells with a transfection marker cut-off of $5x10^3$ mKate MEFL for n = 2 technical replicates. **(b)** Fold repression of gRNA/cognate and non-cognate promoter pairs across different mKate transfection marker cut-offs for at least 2 replicates. Reference fold repression of 1 is represented by the red line. The dashed grey lines represent standard error.

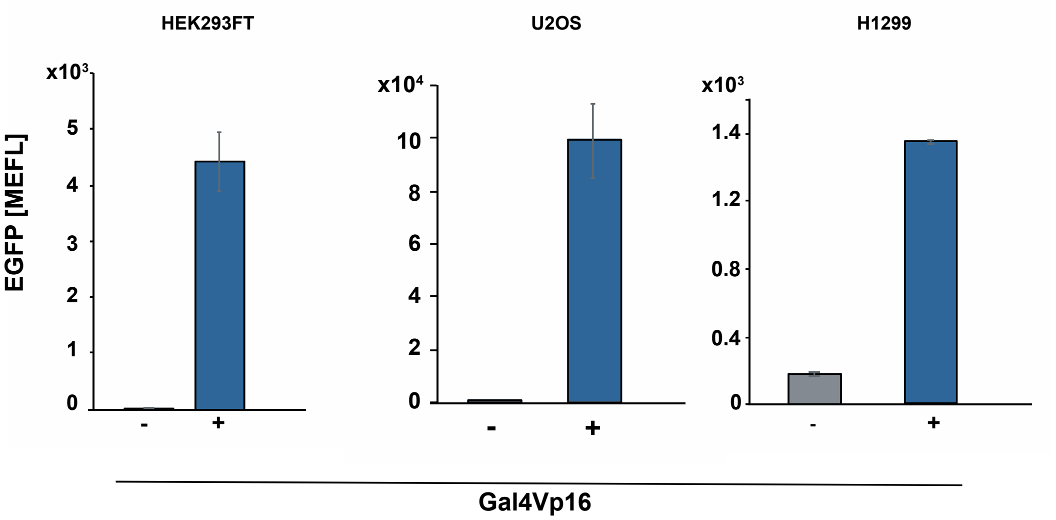

Supplementary Figure 5. Gal4VP16 transcriptional activation. HEK293FT, U2OS and H1299 cell lines show different output expression and background activity in presence and absence of the transcriptional activator. Data represent geometric mean and standard deviation of EGFP MEFL for cells expressing > $2x10^4$ MEFL (HEK293FT, H1299), >$5x10^3$ (U2OS) of transfection marker mKate for at least 2 replicates.

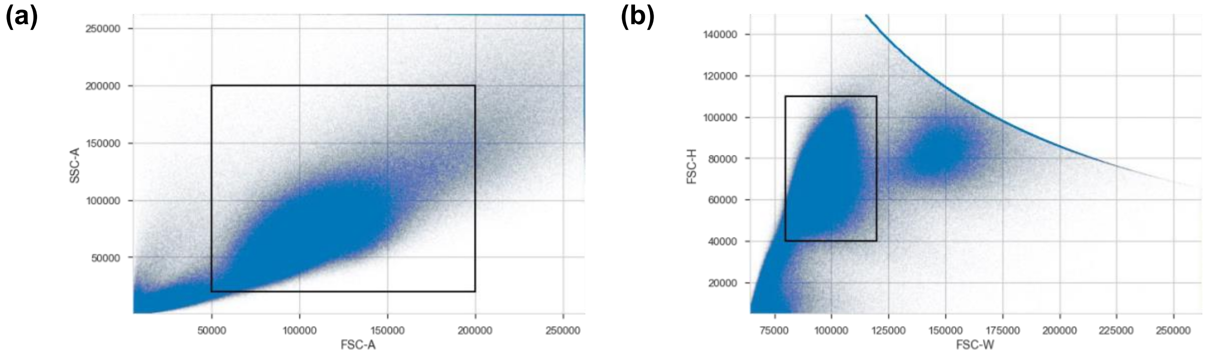

Supplementary Figure 6. Flow Cytometry Gating Strategy (a) Manual Cell Gating for Viable Cells: Cells were manually gated using the Side Scatter (SSC-A) and Forward Scatter (FSC-A) characteristics to select for viable cells. **(b)** Manual Cell Gating for Single Cells: Cells were then gated using their Forward Scatter (FSC-H and FSC-W) properties to select for single cells. The gating is applied for data from HEK293FT cells. The gate vertices for all cells lines are available in the Supplementary Table 4.

Table of resources

REAGENT or RESOURCE	SOURCE	IDENTIFIER
**Bacterial and Virus Strains**		
NEB® 5-alpha Competent *E. coli* (Subcloning Efficiency)	NEB	C2988J
**Chemicals, Peptides, and Recombinant Proteins**		
Phusion Polymerase	NEB	M0530L
T4 PNK	NEB	M0201L
BpiI (BbsI)	ThermoFisher Scientific	ER1011
T4 DNA Ligase	NEB	M0202T
BspMI	NEB	R0502L
DMEM	ThermoFisher Scientific	11960044
Opti-MEM	ThermoFisher Scientific	31985062
Trypsin 0.25%	ThermoFisher Scientific	25200056
DMEM Media – No Phenol Red	ThermoFisher Scientific	31053028
Fetal Bovine Serum, qualified, heat inactivated, E.U.-approved, South America Origin	ThermoFisher Scientific	10500064
Penicillin-Streptomycin (10,000 U/mL)	ThermoFisher Scientific	15140122
L-Glutamine (200 mM)	ThermoFisher Scientific	25030081
MEM Non-Essential Amino Acids Solution (100X)	ThermoFisher Scientific	11140050
Rainbow Calibration Particles, 8 Peaks	Spherotech	RCP-30-5A
Lipofectamine 3000	ThermoFisher Scientific	L3000008

FuGENE® 6 Transfection Reagent	Promega	E2691
**Experimental Models: Cell Lines**		
HEK293 FT		
H1299		
U2OS		
**Oligonucleotides**		
ssDNA oligos (see Supplementary Table 3 for sequences)	IDT	N/A
**Recombinant DNA**		
pcDNA3.1-hLbCpf1	[1]	Addgene #69988
pcDNA3.1-hdLbCas12a	This study	
pSIREN_U6-shRNA_FF5-CMV-iRFP		Courtesy of Dr. Wroblewska
pSIREN_U6-Cas12a gRNA	This study	
pLS1-BoxCDGC_2xKMet_-cloningvector	This study	
pLS1-BoxCDGC_2xKMet_-cloningvector-RFP	This study	
pT-GTW6-CMV-mKate	[2]	mKate: Evrogen[3]
pLS1	[2]	
Gal4-VP16	[2]	
pL-A2	[2]	
pSIREN U6 gRNA Lb541	This study	
pSIREN U6 gRNA Lb1226	This study	
pSIREN U6 gRNA Lb1271	This study	
pSIREN U6 gRNA Lb1674	This study	
pSIREN U6 gRNA Lb120	This study	
pSIREN U6 gRNA Lb512	This study	
pSIREN U6 gRNA Lb1367	This study	
pSIREN U6 gRNA Lb1608	This study	
pSIREN U6 gRNA Lb1675	This study	
pLS1-Lb1674 p1 smCMV	This study	
pLS1-Lb1226 p1 smCMV	This study	
pLS1-Lb1674 p3 smCMV	This study	
pLS1-Lb1226 p3 smCMV	This study	

pLS1-Lb1674 p4 smCMV	This study	
pLS1-Lb1226 p4 smCMV	This study	
pLS1-Lb541 p2 smCMV	This study	
pLS1-Lb1226 p2 smCMV	This study	
pLS1-Lb1271 p2 smCMV	This study	
pLS1-Lb1674 p2 smCMV	This study	
pLS1-Lb120 p2 smCMV	This study	
pLS1-Lb512 p2 smCMV	This study	
pLS1-Lb1367 p2 smCMV	This study	
pLS1-Lb1608 p2 smCMV	This study	
pLS1-Lb1675 p2 smCMV	This study	
**Software and Algorithms**		
R Studio – Open source edition	Rstudio	https://www.rstudio.com/
TASBE	[4]	https://github.com/bpteague/cytoflow
Cas-OFFinder	[5]	https://github.com/snugel/cas-offinder
MELTING	[6,7]	http://www.ebi.ac.uk/biomodels/tools/melting/
MultiRNAFold 2.0		http://www.rnasoft.ca/download/
R2oDNA Designer	[8]	http://www.r2odna.com/
FlowJo	FlowJo, LLC	https://www.flowjo.com/
gDesigner	This study	https://github.com/jmacdona/gDesigner

Supplementary References

1. Zetsche, B. *et al.* Cpf1 Is a Single RNA-Guided Endonuclease of a Class 2 CRISPR-Cas System. *Cell* 163, 759–771 (2015).

2. Wroblewska, L. *et al.* Mammalian synthetic circuits with RNA binding proteins for RNA-only delivery. *Nat Biotechnol* 33, 839–841 (2015).

3. Shcherbo, D. *et al.* Bright far-red fluorescent protein for whole-body imaging. *Nat Methods* 4, 741–746 (2007).

4. Beal, J. *et al.* An End-to-End Workflow for Engineering of Biological Networks from High-Level Specifications. *Acs Synth Biol* 1, 317–331 (2012).

5. Bae, S., Park, J. & Kim, J.-S. Cas-OFFinder: a fast and versatile algorithm that searches for potential off-target sites of Cas9 RNA-guided endonucleases. *Bioinformatics* 30, 1473–1475 (2014).

6. Dumousseau, M., Rodriguez, N., Juty, N. & Novère, N. L. MELTING, a flexible platform to predict the melting temperatures of nucleic acids. *Bmc Bioinformatics* 13, 101–101 (2012).

7. Novère, N. L. MELTING, computing the melting temperature of nucleic acid duplex. *Bioinform Oxf Engl* 17, 1226–7 (2001).

8. Casini, A. *et al.* R2oDNA Designer: Computational Design of Biologically Neutral Synthetic DNA Sequences. *Acs Synth Biol* 3, 525–528 (2014).
